# Transcriptome characterisation, SSR marker development and genetic diversity analysis of the endangered species *Camellia cucphuongensis* Ninh & Rosmann using Illumina sequencing

**DOI:** 10.3897/BDJ.14.e186683

**Published:** 2026-03-31

**Authors:** Bei Cui, Lian Wang, Quoc Khang Tran, Thi Tuyet Xuan Bui, Thi Tuyen Phung, Syed Noor Muhammad Shah, Hieu Yen Ha, Van Thang Bui, Duy Dinh Vu

**Affiliations:** 1 Jiangsu Vocational Institute of Architectural Technology, School of Ecological Engineering, Xuzhou 221000, Jiangsu, China Jiangsu Vocational Institute of Architectural Technology, School of Ecological Engineering, Xuzhou 221000 Jiangsu China; 2 The Olympia schools, To Huu Street, Hanoi, Vietnam The Olympia schools, To Huu Street Hanoi Vietnam; 3 Institute of Biology, Vietnam Academy of Science and Technology, Hanoi, Vietnam Institute of Biology, Vietnam Academy of Science and Technology Hanoi Vietnam; 4 Faculty of Forest Resources and Environmental Management, Vietnam National University of Forestry, Xuan Mai, Hanoi, Vietnam Faculty of Forest Resources and Environmental Management, Vietnam National University of Forestry, Xuan Mai Hanoi Vietnam; 5 Department of Horticulture, Gomal University, Dera Ismail Khan, Pakistan Department of Horticulture, Gomal University Dera Ismail Khan Pakistan; 6 Faculty of Biotechnology, Vietnam National University of Agriculture, Ngo Xuan Quang, Gia Lam, Hanoi, Vietnam, Hanoi, Vietnam Faculty of Biotechnology, Vietnam National University of Agriculture, Ngo Xuan Quang, Gia Lam, Hanoi, Vietnam Hanoi Vietnam; 7 College of Forestry Biotechnology, Vietnam National University of Forestry, Xuan Mai, Hanoi, Vietnam, Hanoi, Vietnam College of Forestry Biotechnology, Vietnam National University of Forestry, Xuan Mai, Hanoi, Vietnam Hanoi Vietnam; 8 Joint Vietnam - Russia Tropical Science and Technology Research Center, 63 Nguyen Van Huyen, Nghia Do, Hanoi, Vietnam Joint Vietnam - Russia Tropical Science and Technology Research Center, 63 Nguyen Van Huyen, Nghia Do Hanoi Vietnam

**Keywords:** *Camellia cucphuongensis*, Illumina HiSeq 4000, transcriptome, EST-SSR, genetic diversity, population structure

## Abstract

Overharvesting for ornamental and medicinal purposes, combined with ongoing habitat loss and fragmentation in Vietnam, has severely threatened wild populations of *Camellia cucphuongensis*. Effective conservation and management of this species, therefore, require robust genomic resources and informative molecular markers to quantify genetic diversity and population structure. In this study, we generated the first transcriptome dataset for *C. cucphuongensis* and developed expressed sequence tag–simple sequence repeat (EST-SSR) markers using Illumina HiSeq™ 4000 sequencing. A total of 13,600,954 clean reads were obtained (Q20 = 97.55%, Q30 = 93.11%, GC = 44.08%). De novo assembly produced 118,552 unigenes with a mean length of 541.2 bp and an N50 of 683 bp. Functional annotation revealed that 52,107 and 25,640 unigenes had significant matches in the Nr and Swiss-Prot databases, respectively. Additionally, 28,007 unigenes were assigned to Gene Ontology terms, 27,968 to KOG categories and 11,959 to 117 KEGG pathways. Mining for simple sequence repeats identified 9,661 EST-SSR loci. From 60 screened primer pairs, 11 polymorphic EST-SSR markers were validated and applied to 60 individuals from three natural populations. Genetic diversity was moderate (*N_E_* = 2.17; *PIC* = 0.548; *H_O_* = 0.46; *H_E_* = 0.50), with most variation occurring within individuals (79%) and 11% amongst populations (*F_ST_* = 0.113; *Nm* = 1.96). Principal coordinate analysis (PCoA), discriminant analysis of principal components (DAPC), STRUCTURE and neighbour-joining (NJ) analyses all indicated detectable population structuring, with population CP showing clearer differentiation relative to LH and TL. Collectively, these transcriptomic resources and EST-SSR markers provide practical tools for genetic monitoring and can support conservation strategies that emphasise habitat protection and maintenance of connectivity to mitigate genetic erosion in this endangered golden camellia.

## Introduction

*Camellia* (Theaceae) is a genus of evergreen shrubs and small trees predominantly found in East and Southeast Asia (Zhao et al. 2017). Estimates of species richness within the genus range from approximately 120 species (Ming and Bartholomew 2007) to nearly 280 species (Gao et al. 2005), reflecting both its taxonomic complexity and ongoing discovery of new taxa. Species of *Camellia* have high economic and cultural value due to their ornamental flowers, caffeine and purine alkaloid content and the extensive use of their seeds for edible oil production and their leaves in the pharmaceutical industry. Vietnam is a centre of diversity for the genus, harbouring more than 75 species, many of which are native or endemic (Nguyen et al. 2020). Despite their economic importance, *Camellia* species are increasingly threatened by overexploitation and habitat loss (Xin et al. 2017). Rapid economic development over recent decades has led to substantial deforestation, forest fragmentation and degradation of natural habitats, severely impacting *Camellia* populations (Mao et al. 2025). As a result, population sizes have declined sharply, raising concerns about genetic erosion (Bosse and van Loon 2022). At present, at least 51 *Camellia* species are listed as threatened at both national and global levels (Beech et al. 2017), highlighting the urgent need for effective conservation strategies informed by genetic data.

*Camellia cucphuongensis* Ninh & Rosmann, a rare golden camellia species, is geographically restricted and highly endangered. It is known to occur in Cuc Phuong National Park (Ninh Binh Province) and in Tan Lac and Lac Thuy districts (Phu Tho Province), Vietnam (Tran 1998), with additional records reported from Yunnan, China (Gao et al. 2005). In Vietnam, the species is mainly confined to secondary forests, where it flowers annually from October to December. Natural populations of *C. cucphuongensis* have experienced severe declines due to habitat conversion for agriculture and urbanisation, as well as intensive harvesting of flower buds for commercial trade. As a result, the species has been assessed as Critically Endangered (CR) under the IUCN Red List Criteria (Orel et al. 2018). Despite its high conservation value, genomic resources for *C. cucphuongensis* remain extremely limited and no comprehensive genetic or population-level studies have been conducted to date.

Understanding the genetic diversity and population structure of endangered species is essential for designing effective conservation and management programmes (Wariss et al. 2025). Microsatellite markers, also known as simple sequence repeats (SSRs), are short tandemly repeated DNA motifs that are ubiquitous in eukaryotic genomes (Vieira et al. 2016). Variation in the number of repeat units generates high levels of allelic polymorphism, making SSRs particularly informative genetic markers. SSRs are characterised by codominant inheritance, high abundance and reproducibility (Vu et al. 2020, Pham et al. 2024, Li et al. 2024, Bui and Vu 2024, Ha et al. 2025, Phan et al. 2026). There are various molecular marker systems, but their use depends on the study's objectives and the target species. For example, Amplified Fragment Length Polymorphisms (AFLPs) and Random Amplified Polymorphic DNA (RAPDs) can produce many markers without prior genomic information. However, the markers are dominant and have limited potential in heterozygosity and population structure analyses (Mueller and Wolfenbarger 1999). Single Nucleotide Polymorphisms (SNPs) are abundant and co-dominant, but their development and genotyping can be expensive. The demand for non-model organisms without a reference genome often requires approaches such as reduced-representation sequencing (Davey et al. 2011). In contrast to other molecular markers, SSRs markers are often species-specific, rendering them especially suitable for analysing intraspecific genetic diversity and population structure (Yang et al. 2025). Consequently, SSRs have been widely applied in studies of plant genetic relationships, population differentiation and conservation genetics (Liu et al. 2013, Fan et al. 2025). A significant knowledge gap exists concerning the genetic basis of environmental adaptation and stress tolerance in *C. cucphuongensis*. Addressing this gap through transcriptome sequencing is a powerful and economical strategy to elucidate the underlying molecular mechanisms and to generate genome-wide markers suitable for population genetic analyses. A transcriptome comprises the complete set of RNA molecules, including messenger RNA (mRNA), ribosomal RNA (rRNA), transfer RNA (tRNA) and other non-coding RNAs, expressed in a cell or tissue under specific environmental conditions or developmental stages (Martínez-Campos et al. 2025). Advances in next-generation sequencing (NGS) technologies, particularly high-throughput RNA sequencing (RNA-Seq), have enabled the rapid generation of large-scale expressed sequence data for non-model plant species (Li et al. 2021).

Transcriptome-based approaches enable the efficient development of numerous unigene-derived SSRs (EST-SSRs), which are valuable tools for genome-wide analyses due to their combination of functional relevance and high polymorphism (Wang et al. 2011, Tan et al. 2013, Tong and Gao 2020, Parmar et al. 2022, Xie et al. 2025). In this study, we used the Illumina HiSeq™ 4000 platform to generate a comprehensive transcriptome dataset for *C. cucphuongensis*. From the assembled unigenes, we developed a large set of EST-SSR markers, which was then used to assess genetic diversity and population structure amongst natural populations in Vietnam. These genomic resources provide a valuable foundation for functional gene annotation, population genetics and evolutionary studies within *Camellia* and offer critical molecular tools to support the conservation and sustainable management of this endangered species.

## Material and methods

### Plant material and RNA extraction

Fresh leaf, stem and root tissues of *C. cucphuongensis* were collected from four wild naturally occurring individuals in Cuc Phuong National Park, Ninh Binh Province, Vietnam (105°35′57″E; 20°21′06″N) in April 2025. Immediately after collection, samples were frozen in liquid nitrogen in the field and transported to the Laboratory of Molecular Biology, Joint Vietnam–Russia Tropical Science and Technology Research Center, where they were stored at −80°C until RNA extraction. Total RNA was isolated separately from each tissue sample using a Plant RNA Kit (Omega Bio-tek, Inc.) following the manufacturer’s instructions, with an additional DNase I treatment to remove genomic DNA contamination. RNA integrity was assessed by electrophoresis on 1% agarose gels and further evaluated using an Agilent 2100 Bioanalyzer (Agilent Technologies, CA, USA). RNA concentration and purity were determined using a NanoDrop ND-2000 spectrophotometer (NanoDrop Technologies, DE, USA). Equal quantities of high-quality RNA from the different tissues were pooled to construct a representative transcriptome for Illumina sequencing, to maximise the diversity of transcripts captured in the reference assembly.

### Library construction and Illumina sequencing

The pooled RNA sample was submitted to Breeding Biotechnologies Co., Ltd. for mRNA library construction and sequencing on the Illumina HiSeq™ 4000 platform (Illumina, Inc., CA, USA). Library preparation was performed using the TruSeq™ RNA Sample Preparation Kit (Illumina, CA, USA) according to the manufacturer’s protocol. Briefly, poly(A)+ mRNA was enriched from total RNA using magnetic oligo(dT) beads (Oligotex mRNA Kit) and fragmented into short segments using an RNA fragmentation kit. First-strand cDNA synthesis was carried out using random hexamer primers and reverse transcriptase (Invitrogen, CA, USA), followed by second-strand cDNA synthesis using DNA polymerase I to generate double-stranded cDNA. Residual RNA was removed using RNase H, after which end repair was performed and a single adenine base was added to the 3′ ends of the cDNA fragments. Sequencing adapters were then ligated to the cDNA fragments. Adapter-ligated fragments were enriched by PCR amplification and PCR products were size-selected by electrophoresis on 2% agarose gels (Certified Low Range Ultra Agarose). Fragments of appropriate size were purified and sequenced on the Illumina HiSeq™ 4000 platform to generate paired-end reads.

### Sequence data processing and de novo transcriptome assembly

Raw sequencing reads were subjected to quality control to remove adapter sequences, reads containing more than 5% ambiguous nucleotides (N) and reads with more than 20% low-quality bases (Phred quality score < 10). High-quality clean reads were retained for subsequent analyses. The filtered reads were de novo assembled into contigs using Trinity version 2.8.5 software with default parameters (Grabherr et al. 2011). To reduce redundancy, the assembled contigs were further clustered into non-redundant unigenes using TGICL version 2.1 (Pertea et al. 2003).

### Functional annotation of unigenes

To infer putative functions, all assembled unigene sequences were compared against several public databases, including the NCBI non-redundant (Nr) protein database (Deng et al. 2006), Swiss-Prot (Apweiler 2004), Gene Ontology (GO) (Ashburner et al. 2000), EuKaryotic Orthologous Groups of proteins (KOG) (Koonin et al. 2004) and Kyoto Encyclopaedia of Genes and Genomes (KEGG) (Kanehisa 2004), using BLASTX with an E-value threshold of ≤ 1 × 10⁻⁵. Protein domain annotation was performed using HMMER software to search against the Pfam database (Finn et al. 2009). GO annotation and functional classification of the unigenes into biological process, molecular function and cellular component categories were conducted using Blast2GO version 5.2 software (Conesa et al. 2005). KOG classification was performed using BLAST searches against the KOG database and KEGG pathway mapping was used to identify metabolic pathways and associated gene functions represented in the transcriptome.

### Development of EST-SSR markers

Microsatellite loci were identified from unigene sequences longer than 1 kb using MISA software (Beier et al. 2017). The minimum repeat thresholds were set as follows: mononucleotide repeats ≥ 12, dinucleotide repeats ≥ 6 and tri-, tetra-, penta- and hexanucleotide repeats ≥ 5. Based on the identified SSR loci, a total of 4,730 EST-SSR primer pairs were designed using Primer3 version 3.0 (Kõressaar et al. 2018) with the following parameters: primer length of 18-24 bp (optimal 20 bp), annealing temperature of 55-65°C (optimal 60°C), GC content of 40-65% (optimal 50%) and expected PCR product size of 100-300 bp. All primers were synthesised by Breeding Biotechnologies Co., Ltd.

### DNA extraction and SSR polymorphism validation

**Figure 1.**
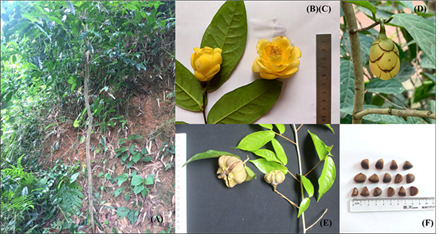
Morphological characteristics of *C. cucphuongensis* Ninh & Rosmann: **A** adult plant in natural habitat; **B** flowering branch; **C** flower in full bloom; **D** flower bud; **E** fruits and leaves; **F** seeds (Photo: Dr. Thi Tuyen Phung).

From the designed primer set, 60 EST-SSR primer pairs were randomly selected for initial PCR screening. Amongst these, 11 primer pairs successfully amplified polymorphic loci across 60 individuals of *C. cucphuongensis* sampled from three wild natural populations: Cuc Phuong (Ninh Binh Province), Tan Lac and Lac Thuy (Phu Tho Province) (Fig. 1, Table 1). Within each population, individuals were sampled at least 300 m apart to reduce the probability of collecting those closely related and to ensure that each sample represented an independent genetic individual. Genomic DNA was extracted from fresh leaf tissue using a Plant DNA Kit (BioTeke, Beijing, China) following the manufacturer’s instructions. DNA quality and concentration were assessed using a NanoDrop ND-2000 spectrophotometer and normalised to 20 ng·µl⁻¹. PCR amplifications were performed in 25 µl reaction volumes containing 2.5 µl template DNA, 12.5 µl 2× Taq Master Mix, 1 µl of each primer and 8 µl nuclease-free water. Amplifications were conducted on a GeneAmp PCR System 9700 (Applied Biosystems, USA) under the following conditions: initial denaturation at 94°C for 3 min; 40 cycles of 94°C for 30 s, primer-specific annealing for 30 s and extension at 70°C for 1 min; followed by a final extension at 72°C for 10 min. PCR products were resolved on 8% polyacrylamide gels in TAE buffer and visualised using GelRed™ nucleic acid gel stain. Fragment sizes were estimated using GenoSens 850 gel analysis software (Clinx Science Instruments Co., Ltd.) with a 35 bp DNA ladder (Invitrogen). Amplicon sizing and quantification were further verified using an Agilent 5300 Fragment Analyzer with a DNF-905 dsDNA kit (Agilent, USA).

### Genetic diversity analyses

Potential genotyping errors and null alleles were assessed using Micro-Checker version 2.0 (van Oosterhout et al. 2004). Polymorphism information content (*PIC*) values were calculated using CERVUS software (Kalinowski et al. 2007). Standard genetic diversity parameters, including number of alleles per locus (*N_A_*), effective number of alleles (*N_E_*), observed heterozygosity (*H_O_*), expected heterozygosity (*H_E_*), inbreeding coefficient (*F_IS_*) and gene flow (*Nm*), were estimated using GenAlEx version 6.5 (Peakall and Smouse 2012). Deviations from Hardy-Weinberg equilibrium (*HWE*) were tested in CERVUS with 1,000 permutations.

### Population structure and phylogenetic analyses

Genetic differentiation amongst populations was quantified using Wright’s fixation index (*F*_*ST*_) (Weir and Cockerham 1984) and G′ST (Hedrick 2005), as implemented in GenAlEx 6.5. The significance of pairwise *F_ST_* values was evaluated using Alequin version 3.1 with 10,000 permutations (Excoffier et al. 2005). Analysis of molecular variance (AMOVA) was also performed in Alequin to partition genetic variation within and amongst populations. Bayesian clustering analysis was conducted using Structure version 2.3.4 (Pritchard et al. 2000) under an admixture model with correlated allele frequencies. Ten independent runs were performed for each value of K (1-15), with 500,000 Markov Chain Monte Carlo (MCMC) iterations following a burn-in period of 100,000 iterations. The optimal number of clusters was determined using Structure Harvester version 0.6.93 (Earl and vonHoldt 2011), based on the ΔK method (Evanno et al. 2005). Results from replicate runs were aligned using Clumpp version 1.1.2 (Jakobsson and Rosenberg 2007) and graphical outputs were generated using Distruct version 1.1 (Rosenberg 2003). Discriminant analysis of principal components (DAPC) was performed using the adegenet package in R version 4.0.2 (Jombart et al. 2010) to identify genetic clusters without prior population information. The optimal number of clusters was determined using the Bayesian Information Criterion (BIC), with K values ranging from 1 to 20. Cross-validation (xvalDAPC) was used to retain the first 14 principal components, explaining 98.5% of the total variance and seven discriminant functions. Phylogenetic relationships amongst individuals were inferred using the neighbour-joining (NJ) method, based on Nei’s genetic distances, implemented in Ntsys version 2.11 (Saitou and Nei 1987) and Mega version 11.0 (Tamura et al. 2021).

## Results

### Illumina sequencing and de novo assembly

Illumina transcriptome sequencing of *C. cucphuongensis* generated a total of 17,379,703 clean reads. After quality filtering, 13,600,954 clean reads were retained, with Q20 = 97.55% and Q30 = 93.11%, respectively. De novo assembly was performed using Trinity, resulting in 1,731,255 contigs with a total length of 166,947,590 bp, an N50 of 155 bp and a mean contig length of 964.3 bp. The majority of contigs (95.41%) were between 0–300 bp in length, while 0.59% ranged from 1,001–2,000 bp. A total of 4,468 contigs exceeded 2,001 bp. From these, 197,570 transcripts were obtained, with a total length of 179,788,275 bp, an N50 of 1,675 bp and a mean length of 910 bp. Amongst these, 24,334 transcripts were longer than 2,001 bp. After redundancy reduction, 118,552 unigenes were generated, with a total length of 64,158,962 bp, an N50 of 683 bp and a mean length of 541.2 bp. Most unigenes fell within the 200-300 bp (44.33%) and 301–500 bp (28.97%) ranges, while 3.82% exceeded 2,001 bp (Table 2).

### Functional annotation and classification of unigenes

**Figure 2.**
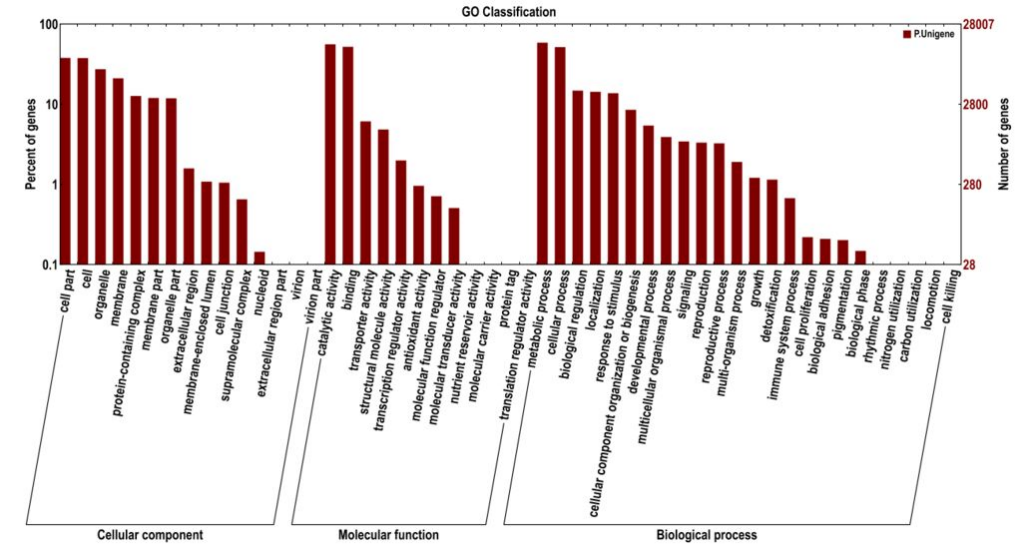
Gene Ontology (GO) classification of *C. cucphuongensis* unigenes into three main categories: biological process, molecular function and cellular component.

**Figure 3.**
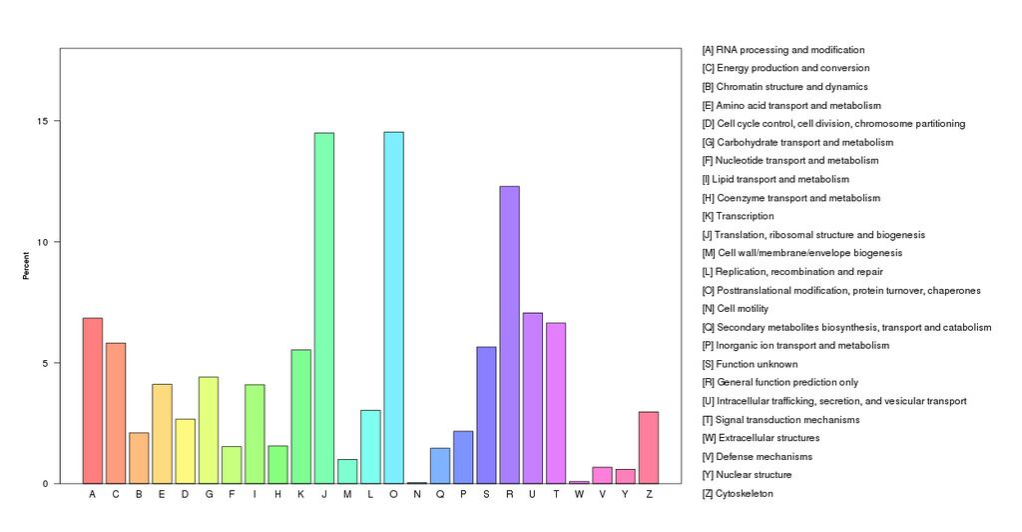
Functional classification of *C. cucphuongensis* unigenes, based on Eukaryotic Orthologous Groups (KOG).

**Figure 4.**
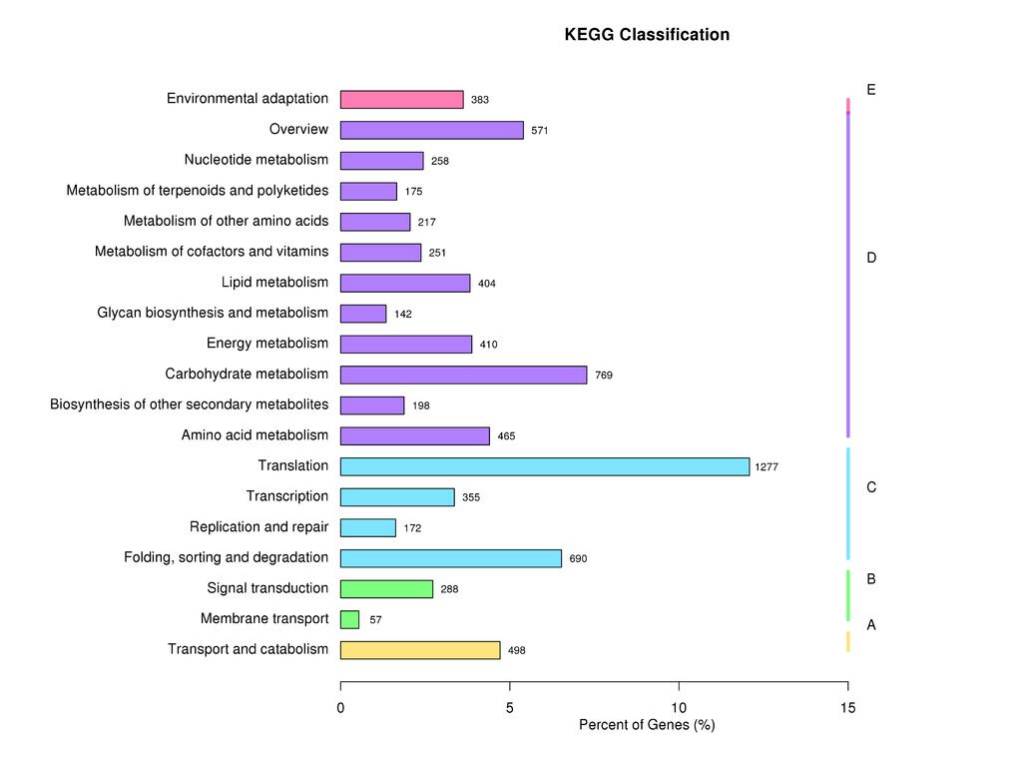
Functional classification of *C. cucphuongensis* unigenes,based on Kyoto Encyclopaedia of Genes and Genomes (KEGG) pathway annotations: Cellular Processes (A); Environmental Information Processing (B); Genetic Information Processing (C); Metabolism (D); Organismal Systems (E).

A total of 52,107, 31,987, 25,640, 28,007, 27,968 and 11,959 unigenes were annotated against the Nr, Pfam, Swiss-Prot, GO, KOG and KEGG databases, respectively (Table 3). Species distribution, based on the Nr database (Suppl. material 1), showed that the top matches were *Vitis vinifera* (7,134), *Dothistroma septosporum* (1,823), *Coffea canephora* (1,445), *Theobroma cacao* (1,317), *Nelumbo nucifera* (1,211), *Sesamum indicum* (1,149), *Ziziphus jujuba* (1,012), *Manihot esculenta* (890), *Citrus sinensis* (748) and *Jatropha curcas* (744). An additional 34,576 unigenes showed similarity to a broad range of other species. GO annotation assigned 28,007 unigenes (23.62%) to three ontologies comprising 51 functional terms (Fig. 2). The biological process category represented the largest proportion (51,939; 42.37%), followed by cellular component (36,389; 29.69%) and molecular function (34,254; 27.94%). Within the cellular component ontology, the most frequently represented terms were cell, cell part, organelle and membrane. In the molecular function ontology, catalytic activity and binding predominated, while, in the biological process, metabolic process and cellular process were the dominant terms. KOG classification assigned 27,968 unigenes to 25 functional categories (Fig. 3). The most abundant category was general function prediction only (5,697; 20.37%), followed by post-translational modification, protein turnover and chaperones (2,912; 10.41%), translation, ribosomal structure and biogenesis (2,263; 8.09%) and signal transduction mechanisms (1,865; 6.67%). KEGG mapping assigned 11,959 unigenes (10.09%) to 117 pathways across five major functional classes (Fig. 4). Metabolism was the largest category (3,860; 50.92%), followed by genetic information processing (2,494; 32.9%), cellular processes (498; 6.57%), organismal systems (383; 5.05%) and environmental information processing (345; 4.55%).

### Frequency and distribution of EST-SSRs

A total of 13,280 sequences (total length 25,815,979 bp) were examined for SSR discovery. In total, 9,661 SSRs were identified; 733 were compound SSRs and 2,261 unigenes contained more than one SSR locus (Table 4). A total of 6,558 sequences contained SSRs. The overall microsatellite frequency in unigenes was 72.75%, with an average density of one SSR per 2.67 kb. SSRs were classified into six repeat types (Suppl. material 2): mononucleotide repeats (3,986; 41.26%) and dinucleotide repeats (3,926; 40.64%) were the most abundant, followed by trinucleotides (1,617; 16.74%), tetranucleotides (102; 1.06%), pentanucleotides (13; 0.13%) and hexanucleotides (17; 0.18%). Across repeat numbers, SSRs with 10 repeats (1,662; 17.2%) were most frequent, followed by those with six repeats (1,415; 14.65%) and five repeats (1,000; 10.35%), whereas each of the remaining repeat­ number classes each represented < 10% of SSRs (Suppl. material 4). Motif composition showed that, amongst mononucleotides, A/T (3,908; 40.45% of total SSRs) dominated, with C/G (78; 0.81%) being rare. Amongst dinucleotides, AG/CT (3,025; 31.31%) was most abundant, followed by AT/AT (655; 6.78%) and AC/GT (244; 2.53%). Amongst trinucleotides, AAG/CTT (415; 4.3%) was the most frequent motif. Amongst tetranucleotides, AAAT/ATTT (30; 0.31%) was the predominant motif, followed by AAAG/CTTT (19; 0.2%). Overall, the 17 most frequent motifs accounted for 99.05% of all SSRs (Suppl. material 3).

### Validation of EST-SSR markers and genetic diversity

Of the 4,730 designed EST-SSR primer pairs, 60 were initially screened across 10 individuals. This identified 11 polymorphic loci and 32 monomorphic loci. The 11 robust polymorphic markers were then used for population genetic analyses. At the species level (N = 60), these 11 loci yielded mean values of *N*_*A*_ = 3.91, *N_E_* = 2.17, *PIC* = 0.548, *H_O_* = 0.46 and *H_E_* = 0.50, with a mean *F_IS_* = 0.07 and *F_IS_IIM* = 0.015 (Tables 1, 5). All three populations showed 100% polymorphic loci (Table 1). Population-level genetic diversity parameters were as follows: CP (*N*_*A*_ = 3.73, *N_E_* = 2.40, *H_O_* = 0.49 and *H_E_* = 0.53), LH (*N*_*A*_ = 4.18, *N_E_* = 2.24, *H_O_* = 0.46 and *H_E_* = 0.54) and TL (*N*_*A*_ = 3.82, *N_E_* = 1.87, *H_O_* = 0.43 and *H_E_* = 0.44). Population-level inbreeding coefficients (*F_IS_*) were 0.04 (CP), 0.16 (LH), and 0.01 (TL), indicating heterozygote deficiency particularly in LH (Table 1).

### Population differentiation, clustering and phylogenetic patterns

**Figure 5.**
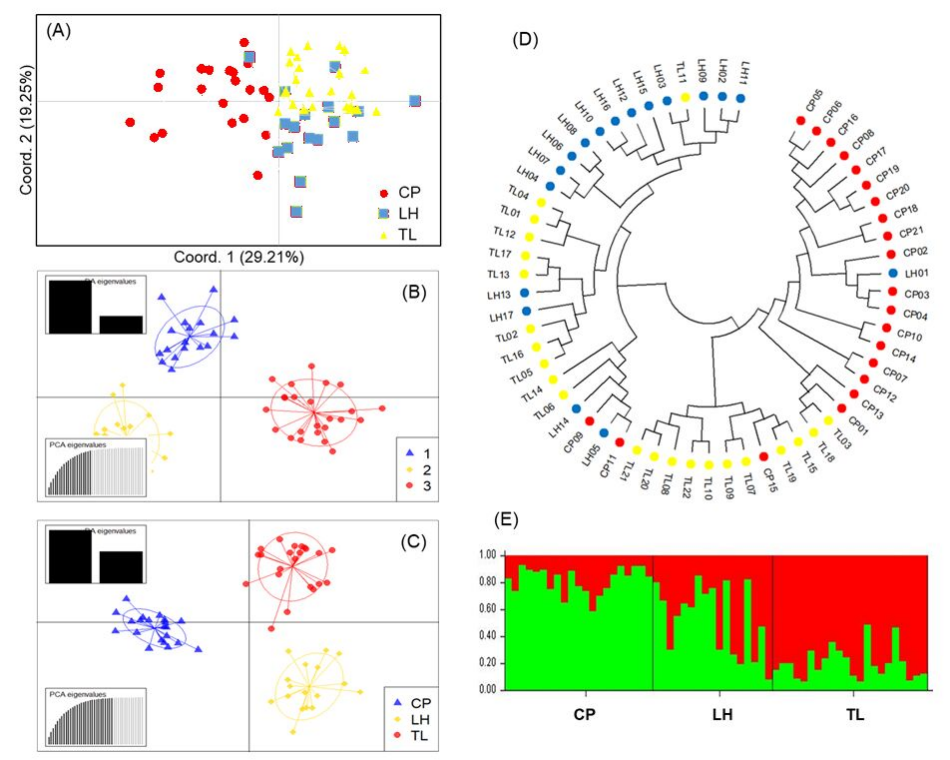
Genetic relationships and population structure of *C. cucphuongensis* inferred from 11 EST-SSR loci. **(A)** Principal coordinate analysis (PCoA) of individuals from three populations (CP, LH, TL); **(B)** DAPC scatter plot inferred without prior population information; **(C)** DAPC scatter plot using prior population information; **(D)** Neighbour-Joining tree based on Nei’s genetic distance; **(E)** Bayesian clustering analysis (STRUCTURE) showing the inferred genetic clusters across populations.

The analysis of molecular variance (AMOVA: 9,999 permutations) revealed that the majority of genetic variation was distributed within individuals (79%), with 11% amongst populations and 10% amongst individuals within populations (Table 6). The *F*_ST_ value was 0.113 (P < 0.05), corresponding to a gene flow (*Nm*) estimate of 1.96 (Table 6). Pairwise comparisons indicated moderate genetic structure amongst the populations. The *F*_ST_ values were 0.114 between CP and LH, 0.138 between CP and TL and 0.081 between LH and TL, with corresponding *N*m values of 1.941, 1.568 and 2.843, respectively (Suppl. material 5). Principal Coordinate Analysis (PCoA) separated individuals primarily along the first axes, which cumulatively explained 64.01% of the variance (Fig. 5A). Discriminant Analysis of Principal Components (DAPC) without prior population information identified three genetic clusters corresponding to geographic origins CP, LH and TL; DAPC incorporating prior information further illustrated assignment patterns within and amongst populations (Fig. 5B and C). Bayesian clustering analysis implemented in STRUCTURE supported *K* = 2 as the most likely number of genetic clusters (ΔK = 312.8). All individuals exhibited admixture between two ancestral components (Fig. 5E). Cluster 1 was most frequent in CP (82.0%), moderate in LH (53.2%) and lowest in TL (20.4%), whereas Cluster 2 showed the opposite pattern, contributing 18.0%, 46.5% and 79.6% in CP, LH and TL, respectively (Fig. 5E). A neighbour-joining tree, based on Nei’s genetic distance, revealed two major branches, but the relationships amongst individuals were not strictly concordant with geographic origin (Fig. 5D). This pattern is consistent with the admixture proportions inferred from STRUCTURE and the overlap observed in ordination analyses.

## Discussion

### Transcriptome assembly quality and comparative evaluation within Camellia

Microsatellites or simple sequence repeats (SSRs) are tandemly repeated motifs of 1–6 nucleotides that are ubiquitously distributed throughout eukaryotic genomes, occurring in both coding and non-coding regions (Tautz and Renz 1984, Charlesworth et al. 1994). Owing to their high levels of polymorphism, codominant inheritance and reproducibility, SSR markers have been widely applied in studies of genetic structure, gene mapping, evolutionary biology and plant breeding (Kumar et al. 2024). In addition to their utility as molecular markers, SSRs may also influence gene regulation and function, thereby contributing to phenotypic variation and adaptive evolution (Ahmad et al. 2018). In the present study, we report the first comprehensive transcriptomic characterisation of *C. cucphuongensis* using next-generation sequencing (NGS). The Illumina HiSeq™ 4000 platform generated 118,552 unigenes, with an N50 length of 683 bp and a mean unigene length of 541 bp, providing a substantial genomic resource for this endangered species. Although these assembly metrics are lower than those reported for *Camellia taliensis* (N50 = 995 bp; mean = 685 bp) (Zhang et al. 2015), *C. oleifera* (N50 = 962 bp; mean = 708 bp) and *C. oleifera* (N50 = 771 bp) (Xia et al. 2014), they exceed those reported for *C. sinensis* (N50 = 480 bp; mean = 402 bp) (Tan et al. 2013). Our N50 is moderate compared to some *Camellia* species, but it is higher than that of *C. sinensis* (480 bp). This shows that high-quality transcriptomic resources can be produced even for rare species with limited samples. The N50 value has a direct influence on the capture ability of full-length transcripts and identifies functional domains. For conservation genomics applications, this assembly quality provides sufficient resolution for marker development and diversity assessment, though it may limit full-length gene discovery. Future conservation efforts that need full gene characterisation would benefit from long-read or deeper sequencing technologies.

### Functional annotation reveals conserved metabolic and regulatory profiles in Camellia

Functional annotation against multiple public databases (Nr, Pfam, Swiss-Prot, GO, KOG and KEGG) provided extensive insights into the functional landscape of the *C. cucphuongensis* transcriptome. GO classification revealed that metabolic and cellular processes were the most abundant terms in the biological process category, while cell and cell part dominated the cellular component category. In the molecular function category, catalytic activity and binding were predominant. This functional distribution aligns with transcriptomic profiles reported for other *Camellia* species, including *C. oleifera* (Ye et al. 2020), *C. chekiangoleosa* (Tian et al. 2022), *C. japonica* (Li et al. 2021) and *C. sinensis* (Parmar et al. 2022). KOG annotation further revealed that the largest functional classes were "general function prediction only" and "post-translational modification, protein turnover and chaperones", closely reflecting patterns observed in *C. taliensis* (Zhang et al. 2015) and *C. oleifera* (Ye et al. 2020). These findings suggest that the transcriptomic architecture of *C. cucphuongensis* is broadly conserved within the genus, with a substantial proportion of genes involved in core cellular maintenance and regulatory processes. The over-representation of "general function prediction only" in KOG classification (20.37%) highlights substantial portions of the transcriptome with unknown functions, which is a common pattern in non-model species. For conservation, this highlights the importance of protecting the species' entire habitat to preserve unknown, but potentially adaptive genetic elements. KEGG pathway analysis highlighted the predominance of pathways related to ribosome functions, oxidative phosphorylation, RNA transport, purine metabolism, glycolysis/gluconeogenesis and the spliceosome, indicating robust transcriptional and metabolic activity. As the metabolic pathway representation is 50.92% of KEGG annotations, it suggests that *C. cucphuongensis* retains full metabolic capacity, despite population decline. This is an encouraging sign for conservation as it shows that genetic erosion has not yet compromised essential physiological functions. Collectively, these results demonstrate that the *C. cucphuongensis* transcriptome encompasses a wide array of molecular functions and biological pathways, providing a strong foundation for understanding the genetic basis of development, metabolism and stress responses in this endangered species. By identifying individuals or populations with unique alleles in these critical pathways, conservation managers can prioritise them for *ex situ* collection or habitat protection, ensuring that the maximum adaptive potential of the species is preserved.

### EST-SSR characteristics and comparison with other Camellia species

A total of 9,661 EST-SSR loci were identified from the *C. cucphuongensis* transcriptome, providing the first SSR resource developed for this species. Mononucleotide and dinucleotide repeats were the most abundant SSR types, followed by trinucleotide repeats. This distribution pattern consistent with those reported for *C. chekiangoleosa* (Tian et al. 2022), *C. japonica* (Li et al. 2021) and *C. sinensis* (Parmar et al. 2022). The strong predominance of A/T-rich motifs, particularly the high frequency of A/T mononucleotide repeats and AG/CT dinucleotide repeats, reflects a significant AT bias in expressed regions, a characteristic widely observed in plant transcriptomes. In contrast, CG/CG motifs were rare in *C. cucphuongensis*, being consistent with previous findings in other *Camellia* species and other angiosperms, likely due to methylation-associated mutation biases in CG dinucleotides. The GC content of *C. cucphuongensis* unigenes (44.08%) was lower than that reported for *C. sinensis* (48%) (Tan et al. 2013), but comparable to values observed in *C. oleifera* (41.51%) (Ye et al. 2020) and *C. oleifera* (39.1%) (Xia et al. 2014). GC content is an important genomic feature influencing DNA stability, gene expression and codon usage bias and interspecific differences may be associated with long-term evolutionary adaptation to distinct ecological niches. The dominance of AAG/CTT amongst trinucleotide repeats and AAAT/ATTT amongst tetranucleotide repeats further supports the notion that SSR motif composition is highly conserved across the genus, suggesting shared evolutionary constraints on microsatellite formation and maintenance.

### Genetic diversity, population structure and evolutionary implications

Using 11 polymorphic EST-SSR markers, moderate genetic diversity was detected at the species level *C. cucphuongensis* (*N_E_* = 2.17, *H_O_* = 0.46, *H_E_* = 0.50). These values are lower than those reported for more widely distributed or less disturbed *Camellia* species, such as *C. taliensis* (*N_E_* = 6.6, *H_O_* = 0.79, *H_E_* = 0.82) (Wang et al. 2023), but comparable to those observed in *C. chekiangoleosa* (Tian et al. 2022), *C. nitidissima* (Li et al. 2019) and C. nitidissima var. phaeopubisperma (Xie et al. 2025). The reduced genetic diversity in *C. cucphuongensis* likely reflects its restricted geographic distribution, small population sizes and long-term anthropogenic pressures, such as habitat loss and overharvesting. AMOVA revealed that 79% of genetic variation was partitioned within individuals, with only 11% occurring amongst populations. This is pattern is typical of perennial, outcrossing woody species (Hamrick and Godt 1990) and is consistent with findings in *C. taliensis* (Wang et al. 2023) and *C. nitidissima* (Li et al. 2019). The moderate overall *F_ST_* (0.09-0.11) and relatively high estimated gene flow (*Nm* = 1.96) suggest that genetic drift has not yet resulted in strong population differentiation, despite spatial separation amongst populations. This gene flow may be facilitated by life-history traits of *Camellia*, including outcrossing mating systems, long lifespan, overlapping generations and effective pollen dispersal. Nevertheless, positive *F_IS_* values across populations indicate heterozygote deficiency, suggesting inbreeding or mating amongst related individuals. This is likely driven by population fragmentation and reduced effective population sizes.

### Population structure analyses and conservation implications

Multivariate (PCoA, DAPC) and Bayesian (STRUCTURE) analyses consistently revealed genetic structuring amongst the three populations (CP, LH and TL). CP formed a genetically distinct unit, while LH and TL showed closer genetic affinity. The partial overlap observed in PCoA and the clear separation in DAPC reflect differences in analytical sensitivity: DAPC being more effective at maximising amongst-group variation. From a conservation perspective, these findings indicate that CP should be considered a distinct management unit. The closer genetic affinity between LH and TL populations may be the influence of natural and anthropogenic factors. There is no direct evidence of transplantation. However, the long history of local activities in the region, for example, traditional uses of Camellia species, may raise the possibility that seed dispersal or modification in the habitat could have influenced current genetic patterns. However, sampling from wild individuals and a moderate gene flow (*Nm* = 2.84 between LH and TL) suggests the gene flow through natural cross-pollination across these populations. It should still be managed separately to preserve local genetic variation. The moderate genetic diversity and ongoing gene flow suggest that *C. cucphuongensis* retains evolutionary potential; however, continued habitat degradation and overexploitation could rapidly erode this genetic reservoir.

## Conclusions

This study provides the first transcriptome resource and EST-SSR marker set for the endangered species *C. cucphuongensis* generated using the Illumina HiSeq™ 4000 platform. A total of 118,552 unigenes were assembled (N50 683 bp, mean length 541 bp) and 9,661 EST-SSRs were identified, enabling the development of informative microsatellite markers for this species. Validation of transcriptome-derived markers yielded 11 polymorphic EST-SSR loci, which were successfully applied to assess genetic diversity and population structure across three wild populations (CP, LH and TL). Overall genetic diversity was moderate (*N_E_* = 2.17; *H_O_* = 0.46; *H_E_* = 0.50; PIC = 0.548), with moderate differentiation amongst populations (*F_ST_* = 0.113; *Nm* = 1.96) and most variation distributed within individuals (79%). Multivariate and Bayesian analyses supported population structuring, with CP showing greater differentiation relative to LH and TL. Collectively, the transcriptome dataset and EST-SSR markers reported here provide practical molecular tools for population monitoring and conservation genetics of *C. cucphuongensis*. Given ongoing habitat loss and overharvesting pressures, conservation actions should prioritise habitat protection and the maintenance of connectivity amongst populations to reduce further genetic erosion.

## Supplementary Material

96E800CE-4EA2-5400-8374-1128BFCC2B31 10.3897/BDJ.14.e186683.suppl1 Supplementary material 1 Species distribution Data type images Brief description Species distribution of Nr-annotated unigenes in *C. cucphuongensis*. File: oo_1562503.png https://binary.pensoft.net/file/1562503 Bei Cui, Lian Wang, Quoc Khang Tran, Thi Tuyet Xuan Bui, Thi Tuyen Phung, Syed Noor Muhammad Shah, Hieu Yen Ha, Van Thang Bui, Dinh Duy Vu

8E9E341E-A7AD-5A30-A9D0-898F56117113 10.3897/BDJ.14.e186683.suppl2 Supplementary material 2 Frequency distribution of EST-SSR repeat motif types Data type images Brief description Frequency distribution of EST-SSR repeat motif types (mono- to hexa-nucleotide repeats) identified from the transcriptome of *C. cucphuongensis*. File: oo_1562504.png https://binary.pensoft.net/file/1562504 Bei Cui, Lian Wang, Quoc Khang Tran, Thi Tuyet Xuan Bui, Thi Tuyen Phung, Syed Noor Muhammad Shah, Hieu Yen Ha, Van Thang Bui, Dinh Duy Vu

388FEC4D-6F4D-5F11-B9B3-5F41C2B87D45 10.3897/BDJ.14.e186683.suppl3 Supplementary material 3 Frequency distribution of SSRs Data type Suppl. material 1 Brief description Frequency distribution of SSRs based on motif types in *C. cucphuongensis* transcriptome. File: oo_1519708.docx https://binary.pensoft.net/file/1519708 Bei Cui, Lian Wang, Quoc Khang Tran, Thi Tuyet Xuan Bui, Thi Tuyen Phung, Syed Noor Muhammad Shah, Hieu Yen Ha, Van Thang Bui, Dinh Duy Vu;

2843EEE6-6E3E-567F-976D-1B063B072D40 10.3897/BDJ.14.e186683.suppl4 Supplementary material 4 Frequency of SSRs based on repeat types Data type etc. Brief description Frequency of SSRs based on repeat types in *C. cucphuongensis* transcriptome. File: oo_1562508.docx https://binary.pensoft.net/file/1562508 Bei Cui, Lian Wang, Quoc Khang Tran, Thi Tuyet Xuan Bui, Thi Tuyen Phung, Syed Noor Muhammad Shah, Hieu Yen Ha, Van Thang Bui, Dinh Duy Vu

C20618F9-525A-5E7E-B54C-5A71F822E35B 10.3897/BDJ.14.e186683.suppl5 Supplementary material 5 Pairwise genetic differentiation Data type etc. Brief description Pairwise genetic differentiation (*F_ST_*) and *Nm* between populations for *C. cucphuongensis* species. File: oo_1562510.docx https://binary.pensoft.net/file/1562510 Bei Cui, Lian Wang, Quoc Khang Tran, Thi Tuyet Xuan Bui, Thi Tuyen Phung, Syed Noor Muhammad Shah, Hieu Yen Ha, Van Thang Bui, Dinh Duy Vu

## Figures and Tables

**Table 1.** Sampling locations and genetic diversity parameters of *C. cucphuongensis* populations, based on 11 EST-SSR loci.

**Population code**	**Locations**	**Latitude (N), Longitude (E)**	**N**	***N_A_***	***N_E_***	***P(%)***	***H_O_***	***H_E_***	***F_IS_***	***F_IS_IIM***
**CP**	Cuc Phuong National Park, Ninh Binh Province	20°21′06″, 105°35′57″	21	3.73	2.40	100	0.49	0.53	0.04	0.012
**LH**	Lac Thuy commune, Phu Tho Province	20°30'25", 105°43'51"	17	4.18	2.24	100	0.46	0.54	0.16	0.020
**TL**	Tan Lac commune, Phu Tho Province	20°33′29″, 105°20′10″	22	3.82	1.87	100	0.43	0.44	0.01	0.013
**Species level**			60	3.91	2.17	100	0.46	0.50	0.07	0.015
Note: *N_A_*, mean number of alleles per locus; *N_E_*, mean number of effective alleles; P(%), percentage of polymorphic loci; *H_O_* and *H_E_*, mean observed and expected heterozygosities, respectively; *F_IS_*, inbreeding coefficient with *p < 0.05. ^∗∗^P < 0.01, ^∗∗∗^P < 0.001; ns, not significant; The individual inbreeding model was performed to evaluate the *F_IS_* index for null allele frequency *F_IS_IIM*.										

**Table 2.** Summary statistics of de novo transcriptome assembly for *C. cucphuongensis*.

**Length range (bp)**	**Unigene**	**Contigs**	**Transcripts**
0-300	52,559	1,651,729	59,627
301-500	34,347	41,773	44,156
501-1000	18,366	23,076	35,986
1001-2000	8,747	10,209	33,467
> 2000	4,533	4,468	24,334
**Total Number**	118,552	1,731,255	197,570
**Total Length**	64,158,962	16,6947,590	179,788,275
**N50 Length**	683	155	1,675
**Mean Length**	541.2	1,651,729	59,627

**Table 3.** Functional annotation of *C. cucphuongensis* in different databases.

**Annotated database**	**Annotated**	**300–1000 (bp)**	≥ **1000(bp)**
**COG**	17,454	6,607	4,696
**GO**	28,007	11,717	6,703
**KEGG**	11,959	5,192	2,881
**KOG**	27,968	11,923	7,350
**Pfam**	31,987	13,156	9,943
**Swissprot**	25,640	11,332	7,797
**Nr**	52,107	22,648	11,811
**All**	53,845	23,182	11,836

**Table 4.** Summary of analyses of expressed sequence Tag–Simple Sequence repeat (EST-SSRs) in *C. cucphuongensis*.

**Item**	**Parameters**	**Number**
EST-SSR	Total number of sequences examined	13,280
	Total size of examined sequences (bp)	25,815,979
	Total number of identified SSRs	9,661
	Number of SSR containing sequences	6,558
	Number of sequences containing more than 1 SSR	2,261
	Number of SSRs present in compound formation	733

**Table 5.** Genetic parameters in eleven SSR loci for *C. cucphuongensis*.

**Locus**	**Sequence of primer (5'–3')**	**Motif type**	**Size (bp)**	***N*_*A*_**	***N_E_***	**Null allele**	***PIC***	***H_O_***	***H_E_***	***F_IS_***	***F_IT_***	***F_ST_***	***P*_HWE_**
**SSR01**	F: AGA TAA AAG ACC CAC GTC AAG G R: GCA ACT CCA AAA CAC ACT CAT T	(TA)9	126-144	5.00	2.04	No	0.909	0.48	0.52	-0.09	-0.04	0.05	ND
**SSR 02**	F: AAT ACG AAC ACG TTG AGA GCA A R: TTC AGT TCC AAT CCA AGC G	(CAC)6	252-264	3.67	2.09	Yes	0.488	0.50	0.20	0.59	0.61	0.05	NS
**SSR03**	F: ACA GCC AAA ATC AAT CCC TAG A R: ACA ACA CAA CAC AAC ACA GTC G	(AG)7	262-298	4.33	2.79	Yes	0.493	0.64	0.32	0.50	0.55	0.09	***
**SSR04**	F: ACT ACC TCC AGC AAT GGA TCT C R: GAC CGT GTA ATT CGG GTT CTT A	(CT)6	192-220	4.67	2.50	No	0.680	0.58	0.62	-0.06	-0.01	0.05	*
**SSR05**	F: AAA AGA GAG AGA GGA TGG ACC C R: TAA CCC TTC AAT TTC CCT TCA C	(AG)6	198-216	3.00	1.63	No	0.570	0.33	0.40	-0.23	-0.13	0.08	NS
**SSR06**	F: TTA TCT TCC TCA AGC TCT CCC TC R: GTT GAA ACG CAG AGG TGG TAA	(CT)8	200-216	2.67	1.80	No	0.334	0.41	0.43	-0.05	0.10	0.14	ND
**SSR07**	F: TCT CTC TCT CTC GGT TTA GGG TT R: GCT TGC TTG ATC TCC TTG ATG	(CT)7	238-259	4.67	2.02	Yes	0.437	0.49	0.42	0.13	0.28	0.18	NS
**SSR08**	F: CCT CTC CTC CCT CCT CCT T R: GAC AAA CAA AGA ATC CAA AGC C	(TCC)7	267-282	3.67	2.28	Yes	0.549	0.50	0.42	0.16	0.26	0.11	*
**SSR09**	F: CTG GAA AAC TGT CAA AAC CAA C R: TGC CTC TTT TAT ATC TGT CTC CTT C	(AG)7	182-202	4.00	2.39	No	0.538	0.56	0.62	-0.10	0.05	0.13	NS
**SSR10**	F: CAG CCA TTG ATG TTG ATG ACT T R: TCC AAA ACC AGA AAG CCT AGA A	(TC)8	146-170	3.67	1.87	No	0.590	0.45	0.47	-0.04	0.04	0.08	NS
**SSR11**	F: TCAACATGGAACTCCCACAA R: TGATGCTTTGTGATGGATGG	(CAG)10	196-258	3.67	2.46	No	0.435	0.59	0.63	-0.07	0.02	0.08	NS
**Mean**				**3.91**	**2.17**		**0.548**	**0.46**	**0.5**	**0.07**	**0.16**	**0.09**	
Note: number of alleles (*N_A_*), effective alleles (*N_E_*), polymorphism information content (*PIC*), observed heterozygosity (*H_O_)*, expected heterozygosity (*HE)*, inbreeding coefficient (*F_IS_*), Null allele the average null allele frequency, coefficient of total inbreeding (*F_IT_*), genetic differentiation index of Weir and Cockerham (*F_ST_*), ns=not significant, *P < 0.05, **P < 0.01, ***P < 0.001													

**Table 6.** Analysis of molecular variance from natural populations for *C. cucphuongensis*.

	**df**	**Sum of squares**	**Variance components**	**Total** **variation (%)**	**F-statistics**
Amongst populations	2	35.081	0.362	11	*F_ST_* = 0.113*** F_IS_ = 0.112*** F_IT_ = 0.213*** Nm = 1.96
Amongst individuals within populations	57	179.644	0.317	10	
Within individuals	60	151.000	2.517	79	
Total	119	365.725	3.196	100	
Note: coefficient within population (*F_IS_*); coefficient of the total population (*F_IT_*); coefficient of differentiation (*F_ST_*); Gene flow (*Nm*). df: Degrees of freedom; *P < 0.05, ^∗∗^P < 0.01, ***p < 0.001.					
