# Supplementary figures and images for: Transcriptome characterisation, SSR marker development and genetic diversity analysis of the endangered species Camellia cucphuongensis Ninh & Rosmann using Illumina sequencing
Source: Biodivers Data J. 2026 Mar 31;14:e186683. doi: 10.3897/BDJ.14.e186683 (PMC13058598; doi:10.3897/BDJ.14.e186683)

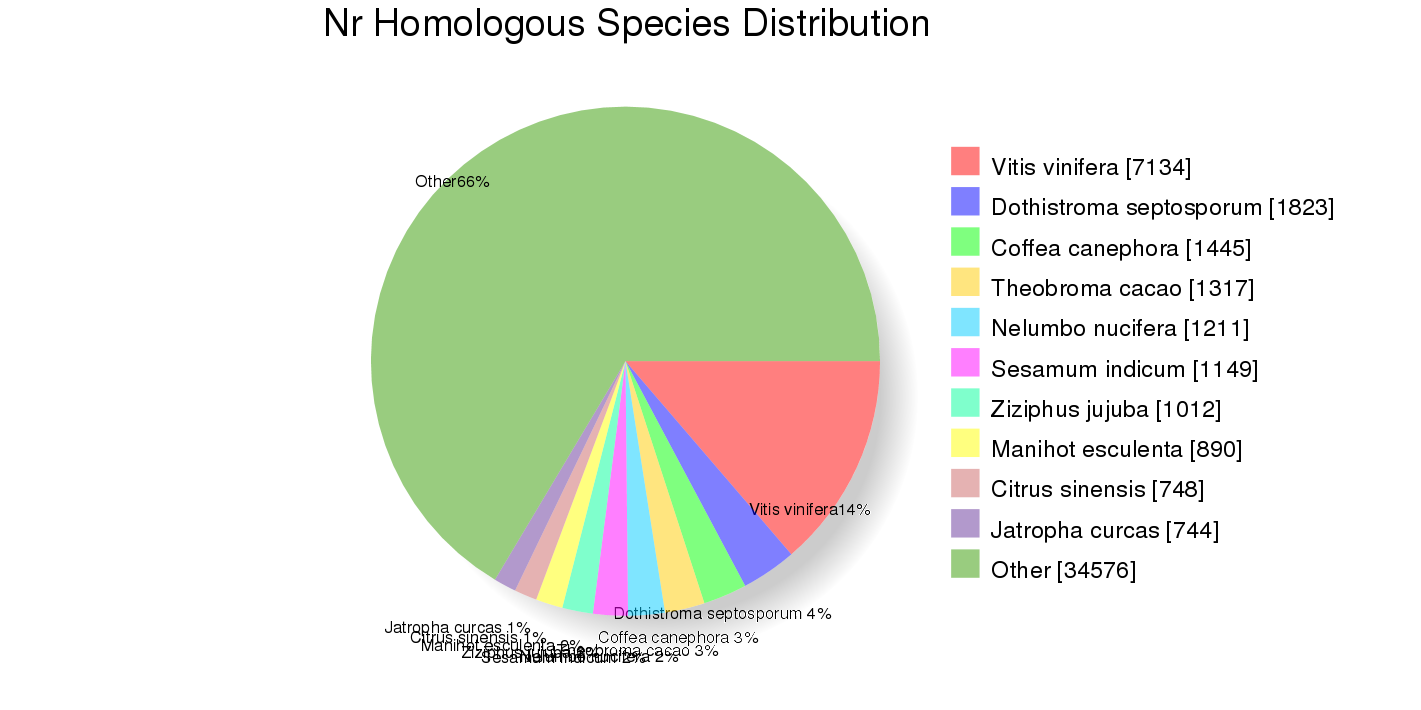

Supplement: Supplementary material 1 — Species distribution [file bdj-14-e186683-s001.png]

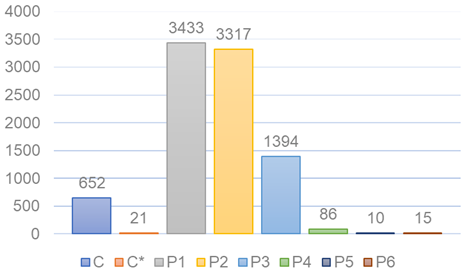

Supplement: Supplementary material 2 — Frequency distribution of EST-SSR repeat motif types [file bdj-14-e186683-s002.png]
