# Supplementary material for: Transcriptome characterisation, SSR marker development and genetic diversity analysis of the endangered species Camellia cucphuongensis Ninh & Rosmann using Illumina sequencing
Source: Biodivers Data J. 2026 Mar 31;14:e186683. doi: 10.3897/BDJ.14.e186683 (PMC13058598; doi:10.3897/BDJ.14.e186683)
Supplement: Supplementary material 3 — Frequency distribution of SSRs [file bdj-14-e186683-s003.docx]

**Table S1.** Frequency distribution of SSRs based on motif types in *C. cucphuongensis* transcriptome

| **Repeats** | **5** | **6** | **7** | **8** | **9** | **10** | **11** | **12** | **13** | **14** | **15** | **16** | **17** | **18** | **19** | **20** | **21** | **22** | **23** | **Total** |
| --- | --- | --- | --- | --- | --- | --- | --- | --- | --- | --- | --- | --- | --- | --- | --- | --- | --- | --- | --- | --- |
| **A/T** | - | - | - | - | - | 1111 | 668 | 392 | 324 | 237 | 191 | 200 | 180 | 175 | 171 | 148 | 80 | 23 | 8 | 3908 |
| **C/G** | - | - | - | - | - | 18 | 14 | 7 | 5 | 5 | 7 | 2 | 6 | 4 | 1 | 2 | 3 | 1 | 3 | 78 |
| **AC/GT** | - | 84 | 44 | 35 | 31 | 28 | 21 |  |  |  |  |  |  | 1 |  |  |  |  |  | 244 |
| **AG/CT** | - | 689 | 548 | 579 | 735 | 406 | 64 | 3 |  | 1 |  |  |  |  |  |  |  |  |  | 3025 |
| **AT/AT** | - | 195 | 106 | 107 | 125 | 96 | 24 | 2 |  |  |  |  |  |  |  |  |  |  |  | 655 |
| **CG/CG** | - | 2 |  |  |  |  |  |  |  |  |  |  |  |  |  |  |  |  |  | 2 |
| **AAC/GTT** | 86 | 28 | 27 | 1 |  | 1 |  |  |  |  |  |  |  |  |  |  |  |  |  | 143 |
| **AAG/CTT** | 212 | 116 | 85 | 2 |  |  |  |  |  |  |  |  |  |  |  |  |  |  |  | 415 |
| **AAT/ATT** | 86 | 35 | 17 |  |  |  |  |  |  |  |  |  |  |  |  |  |  |  |  | 138 |
| **ACC/GGT** | 148 | 67 | 40 | 3 |  |  |  |  |  |  |  |  |  |  |  |  |  |  |  | 258 |
| **ACG/CGT** | 22 | 5 | 6 | 2 |  |  |  |  |  |  |  |  |  |  |  |  |  |  |  | 35 |
| **ACT/AGT** | 20 | 9 | 3 | 2 |  |  |  |  |  |  |  |  |  |  |  |  |  |  |  | 34 |
| **AGC/CTG** | 63 | 35 | 10 | 4 |  | 1 |  |  |  |  |  |  |  |  |  |  |  |  |  | 113 |
| **AGG/CCT** | 97 | 47 | 26 | 4 |  | 1 |  |  |  |  |  |  |  |  |  |  |  |  |  | 175 |
| **ATC/ATG** | 127 | 65 | 35 | 2 |  |  |  |  |  |  |  |  |  |  |  |  |  |  |  | 229 |
| **CCG/CGG** | 50 | 17 | 7 | 3 |  |  |  |  |  |  |  |  |  |  |  |  |  |  |  | 77 |
| **AAAC/GTTT** | 9 | 1 |  |  |  |  |  |  |  |  |  |  |  |  |  |  |  |  |  | 10 |
| **AAAG/CTTT** | 14 | 5 |  |  |  |  |  |  |  |  |  |  |  |  |  |  |  |  |  | 19 |
| **AAAT/ATTT** | 27 | 3 |  |  |  |  |  |  |  |  |  |  |  |  |  |  |  |  |  | 30 |
| **AACC/GGTT** | 1 |  |  |  |  |  |  |  |  |  |  |  |  |  |  |  |  |  |  | 1 |
| **AACG/CGTT** | 1 |  |  |  |  |  |  |  |  |  |  |  |  |  |  |  |  |  |  | 1 |
| **AAGG/CCTT** |  |  | 1 |  |  |  |  |  |  |  |  |  |  |  |  |  |  |  |  | 1 |
| **AATC/ATTG** | 4 | 2 |  |  |  |  |  |  |  |  |  |  |  |  |  |  |  |  |  | 6 |
| **AATG/ATTC** | 2 |  |  |  |  |  |  |  |  |  |  |  |  |  |  |  |  |  |  | 2 |
| **ACAG/CTGT** | 2 |  |  |  |  |  |  |  |  |  |  |  |  |  |  |  |  |  |  | 2 |
| **ACAT/ATGT** | 5 |  |  |  |  |  |  |  |  |  |  |  |  |  |  |  |  |  |  | 5 |
| **ACTC/AGTG** | 3 |  | 1 |  |  |  |  |  |  |  |  |  |  |  |  |  |  |  |  | 4 |
| **ACTG/AGTC** | 1 |  |  |  |  |  |  |  |  |  |  |  |  |  |  |  |  |  |  | 1 |
| **AGAT/ATCT** | 4 | 1 |  |  |  |  |  |  |  |  |  |  |  |  |  |  |  |  |  | 5 |
| **AGCG/CGCT** | 4 | 1 |  |  |  |  |  |  |  |  |  |  |  |  |  |  |  |  |  | 5 |
| **AGGG/CCCT** | 4 | 2 |  |  |  |  |  |  |  |  |  |  |  |  |  |  |  |  |  | 6 |
| **ATCC/ATGG** | 2 |  |  |  |  |  |  |  |  |  |  |  |  |  |  |  |  |  |  | 2 |
| **ATCG/ATCG** | 2 |  |  |  |  |  |  |  |  |  |  |  |  |  |  |  |  |  |  | 2 |
| **AAAAC/GTTTT** | 1 |  |  |  |  |  |  |  |  |  |  |  |  |  |  |  |  |  |  | 1 |
| **AAAAG/CTTTT** |  | 1 |  |  |  |  |  |  |  |  |  |  |  |  |  |  |  |  |  | 1 |
| **AAACC/GGTTT** |  |  |  | 1 |  |  |  |  |  |  |  |  |  |  |  |  |  |  |  | 1 |
| **AAGAG/CTCTT** | 1 |  |  |  |  |  |  |  |  |  |  |  |  |  |  |  |  |  |  | 1 |
| **AAGCT/AGCTT** | 1 |  |  |  |  |  |  |  |  |  |  |  |  |  |  |  |  |  |  | 1 |
| **AATGC/ATTGC** | 1 |  |  |  |  |  |  |  |  |  |  |  |  |  |  |  |  |  |  | 1 |
| **ACACC/GGTGT** | 1 |  |  |  |  |  |  |  |  |  |  |  |  |  |  |  |  |  |  | 1 |
| **ACAGC/CTGTG** | 1 |  |  |  |  |  |  |  |  |  |  |  |  |  |  |  |  |  |  | 1 |
| **ACGAG/CGTCT** | 1 |  |  |  |  |  |  |  |  |  |  |  |  |  |  |  |  |  |  | 1 |
| **AGATC/ATCTG** |  | 1 |  |  |  |  |  |  |  |  |  |  |  |  |  |  |  |  |  | 1 |
| **AGGGG/CCCCT** | 1 |  |  |  |  |  |  |  |  |  |  |  |  |  |  |  |  |  |  | 1 |
| **ATCCC/ATGGG** | 1 |  |  |  |  |  |  |  |  |  |  |  |  |  |  |  |  |  |  | 1 |
| **ATCCG/ATCGG** | 1 |  |  |  |  |  |  |  |  |  |  |  |  |  |  |  |  |  |  | 1 |
| **AAAGCC/CTTTGG** |  | 1 |  |  |  |  |  |  |  |  |  |  |  |  |  |  |  |  |  | 1 |
| **AACAGC/CTGTTG** | 1 |  |  |  |  |  |  |  |  |  |  |  |  |  |  |  |  |  |  | 1 |
| **AACCAG/CTGGTT** | 1 |  |  |  |  |  |  |  |  |  |  |  |  |  |  |  |  |  |  | 1 |
| **AAGCAG/CTGCTT** | 1 |  |  |  |  |  |  |  |  |  |  |  |  |  |  |  |  |  |  | 1 |
| **AAGCCC/CTTGGG** | 1 |  |  |  |  |  |  |  |  |  |  |  |  |  |  |  |  |  |  | 1 |
| **AAGTGG/ACTTCC** |  | 1 |  |  |  |  |  |  |  |  |  |  |  |  |  |  |  |  |  | 1 |
| **AATATG/ATATTC** | 1 |  |  |  |  |  |  |  |  |  |  |  |  |  |  |  |  |  |  | 1 |
| **AATTCC/AATTGG** | 1 |  |  |  |  |  |  |  |  |  |  |  |  |  |  |  |  |  |  | 1 |
| **ACACGC/CGTGTG** |  | 1 |  |  |  |  |  |  |  |  |  |  |  |  |  |  |  |  |  | 1 |
| **ACCAGG/CCTGGT** | 1 |  |  |  |  |  |  |  |  |  |  |  |  |  |  |  |  |  |  | 1 |
| **ACCGCC/CGGTGG** |  |  | 1 |  |  |  |  |  |  |  |  |  |  |  |  |  |  |  |  | 1 |
| **ACCGGC/CCGGTG** | 1 |  |  |  |  |  |  |  |  |  |  |  |  |  |  |  |  |  |  | 1 |
| **ACCTGC/AGGTGC** | 1 |  |  |  |  |  |  |  |  |  |  |  |  |  |  |  |  |  |  | 1 |
| **ACTCCC/AGTGGG** | 1 |  |  |  |  |  |  |  |  |  |  |  |  |  |  |  |  |  |  | 1 |
| **AGAGGG/CCCTCT** | 1 |  |  |  |  |  |  |  |  |  |  |  |  |  |  |  |  |  |  | 1 |
| **AGCATG/ATGCTC** |  |  |  | 1 |  |  |  |  |  |  |  |  |  |  |  |  |  |  |  | 1 |
| **AGCCCC/CTGGGG** |  | 1 |  |  |  |  |  |  |  |  |  |  |  |  |  |  |  |  |  | 1 |
