# Supplementary material for: Transcriptome characterisation, SSR marker development and genetic diversity analysis of the endangered species Camellia cucphuongensis Ninh & Rosmann using Illumina sequencing
Source: Biodivers Data J. 2026 Mar 31;14:e186683. doi: 10.3897/BDJ.14.e186683 (PMC13058598; doi:10.3897/BDJ.14.e186683)
Supplement: Supplementary material 4 — Frequency of SSRs based on repeat types [file bdj-14-e186683-s004.docx]

| **Table 5.** Frequency of SSRs based on repeat types in *C. cucphuongensis* transcriptome | | | | | | | |
| --- | --- | --- | --- | --- | --- | --- | --- |
| **Number of repeat** | **Repeat type** | | | | | | **Total** |
|  | **Mono-** | **Di-** | **Tri-** | **Tetra-** | **Penta-** | **Hexa-** |  |
| 5 | 0 | 0 | 894 | 85 | 10 | 11 | 1,017 |
| 6 | 0 | 970 | 424 | 15 | 2 | 4 | 1,415 |
| 7 | 0 | 698 | 256 | 2 | 0 | 1 | 957 |
| 8 | 0 | 721 | 23 | 0 | 1 | 1 | 746 |
| 9 | 0 | 891 | 0 | 0 | 0 | 0 | 891 |
| 10 | 1,129 | 530 | 3 | 0 | 0 | 0 | 1,662 |
| 11 | 682 | 109 | 0 | 0 | 0 | 0 | 791 |
| 12 | 399 | 5 | 0 | 0 | 0 | 0 | 404 |
| 13 | 329 | 0 | 0 | 0 | 0 | 0 | 329 |
| 14 | 242 | 1 | 0 | 0 | 0 | 0 | 243 |
| 15 | 198 | 0 | 0 | 0 | 0 | 0 | 198 |
| 16 | 202 | 0 | 0 | 0 | 0 | 0 | 202 |
| 17 | 186 | 0 | 0 | 0 | 0 | 0 | 186 |
| 18 | 179 | 1 | 0 | 0 | 0 | 0 | 180 |
| 19 | 172 | 0 | 0 | 0 | 0 | 0 | 172 |
| 20 | 150 | 0 | 0 | 0 | 0 | 0 | 150 |
| 21 | 83 | 0 | 0 | 0 | 0 | 0 | 83 |
| 22 | 24 | 0 | 0 | 0 | 0 | 0 | 24 |
| 23 | 11 | 0 | 0 | 0 | 0 | 0 | 11 |
| **Total** | 3,986 | 3,926 | 1,617 | 102 | 13 | 17 | 9,661 |
