# Supplementary material for: Transcriptome characterisation, SSR marker development and genetic diversity analysis of the endangered species Camellia cucphuongensis Ninh & Rosmann using Illumina sequencing
Source: Biodivers Data J. 2026 Mar 31;14:e186683. doi: 10.3897/BDJ.14.e186683 (PMC13058598; doi:10.3897/BDJ.14.e186683)
Supplement: Supplementary material 5 — Pairwise genetic differentiation [file bdj-14-e186683-s005.docx]

| **Table 8.** Pairwise genetic differentiation (Fst) and Nm between populations for *C. cucphuongensis* species | | | |
| --- | --- | --- | --- |
|  | **CP** | **LH** | **TL** |
| **CP** | 0.000 | 1.941 | 1.568 |
| **LH** | 0.114 | 0.000 | 2.843 |
| **TL** | 0.138 | 0.081 | 0.000 |
| *Note: Fst Values below diagonal; Nm Values above diagonal* | | | |
